# The role of clinical factors and immunocheckpoint molecules in the prognosis of patients with supratentorial extraventricular ependymoma: a single-center retrospective study

**DOI:** 10.1007/s00432-020-03425-1

**Published:** 2021-01-02

**Authors:** Liguo Wang, Song Han, Changxiang Yan, Yakun Yang, Zhiqiang Li, Zuocheng Yang

**Affiliations:** 1 Department of Neurosurgery, Fuxing Hospital, Capital Medical University, Beijing, 100038 China; 2 Department of Neurosurgery, Sanbo Brain Hospital, Capital Medical University, Beijing, 100093 China

**Keywords:** Supratentorial extraventricular ependymoma (SEE), Prognosis, Programmed death ligand-1 (PD-L1), Extent of resection (EOR), Ki-67, Neural cell adhesion molecule L1 (L1CAM)

## Abstract

**Purpose:**

Supratentorial extraventricular ependymoma (SEE) is a rare subset of ependymomas located in the supratentorial parenchyma, and little is known regarding its management and prognosis. Our study aimed to reveal the prognostic factors in patients with SEE and the roles of programmed death ligand-1 (PD-L1), programmed cell death protein 1 (PD-1), Ki-67, and neural cell adhesion molecule L1 (L1CAM) in predicting these patients’ outcomes.

**Methods:**

We retrospectively studied the clinical features and prognostic factors in 48 patients with SEE admitted to our center from April 2008 to October 2018. Tissue slides were constructed from patient samples, and PD-L1, PD-1, Ki-67, and L1CAM expression levels were evaluated by immunohistochemistry.

**Results:**

Patients with gross total resection (GTR) had better progression-free survival than patients with subtotal resection (STR). Moreover, the recurrence hazard ratios in patients with STR at 3, 5, and 10 years were 8.746, 6.866 and 3.962 times those of patients with GTR, respectively. PD-L1 positivity predicted worse progression-free survival, while the recurrence hazard ratios for patients with PD-L1 positivity at 3, 5, and 10 years were 10.445, 5.539, and 3.949 times those of patients with PD-L1 negativity, respectively. Multivariate analysis revealed that PD-L1 expression and GTR could independently predict outcomes in patients with SEE.

**Conclusion:**

PD-L1 expression was an independent and more readily obtained predictor of outcomes, representing a simple and reliable biological prognostic factor for patients with SEE. Further studies are needed to explore PD-L1 inhibitor treatment for patients with ependymoma.

**Clinical trial registration:**

No clinical trials were performed in the study.

## Introduction

Ependymomas are rare neoplasms of the central nervous system (CNS), accounting for 3.14% of all CNS tumors or 3–9% of all intracranial glial neoplasms (Chen et al. 2013). Among intracranial ependymomas, 60% are infratentorial, and 40% are supratentorial (Leng et al. 2016). Supratentorial extraventricular ependymoma (SEE) occurs outside the ventricular system of the brain and accounts for approximately 50% of supratentorial ependymomas (Wang et al. 2018).

The reported prognostic factors of ependymoma include age, tumor location, histological grade, the extent of resection (EOR), metastatic spread, Ki-67 status, and adjuvant radiotherapy (Metellus et al. 2007; Kuncova et al. 2009; Merchant et al. 2009; Pejavar et al. 2011; Tarapore et al. 2013; Sayegh et al. 2014). EOR is the most essential prognostic factor for ependymoma (Venkatramani et al. 2013; Lin and Chintagumpala 2015; Ye et al. 2015; Ramaswamy and Taylor 2016; Sato et al. 2017; Jung et al. 2018; Svoboda et al. 2018). The Ki-67 protein is a biomarker for proliferation that often correlates with the clinical course of malignant tumors (Pérez-Ramírez et al. 2019).

Advances in clinical molecular biology have provided new insights into the histology and genotyping of ependymomas. Nine molecular subgroups were identified by DNA methylation analysis, three of which were located at supratentorial sites (Pajtler et al. 2015). The subtype (ependymoma, RELA fusion-positive) was included in the identification of ependymas for the first time in the classification of CNS tumors by the WHO in 2016 (Reni et al. 2017). Neural cell adhesion molecule L1 (L1CAM), one of the protein products of the RELA fusion gene, is of interest, and Nambirajan and Witt reported that programmed death ligand-1 (PD-L1) is upregulated in ST-RELA ependymomas (Witt et al. 2018; Nambirajan et al. 2019). In this study, we aimed to investigate the prognostic factors in patients with SEE and the roles of PD-L1, programmed death receptor-1 (PD-1), Ki-67, and L1CAM in predicting these patients’ outcomes.

## Materials and methods

### Patients

This study was a retrospective study involving a series of 48 patients with SEE who underwent surgery between April 2008 and October 2018 at the Sanbo Brain Hospital, Capital Medical University, Beijing, China and were enrolled. All cases were independently rereviewed by a senior pathologist, and the histological diagnoses were confirmed according to 2016 World Health Organization (WHO) Classification of Central Nervous System Tumors. The clinical data, including age at diagnosis, sex, treatment, and pathology results, were obtained from the medical records of the enrolled patients. The evaluation of the EOR was based on postoperative contrast MRI. The EOR was recorded as gross total resection (GTR) if no tumor was apparent on the postoperative MRI. Subtotal resection (STR) was defined as residual tumor. Moreover, we collected recurrence features, including time, location, and distribution of histological grades.

### Follow-up

The follow-up data were obtained from the clinical records, and the date of death was obtained from medical records and telephone interviews. Seven (14.58%) patients were lost to follow-up. Overall survival (OS) was determined as the time from the date of the initial diagnosis at presentation to the date of patient death. Progression-free survival (PFS) was determined as the time from the date of the first operation to the date of recurrence or tumor progression. OS and PFS were the primary endpoints of this study.

This study was reviewed and deemed exempt by our ethical review board. The BioBank protocols are in accordance with the ethical standards of our institution and with the 1964 Helsinki declaration and its later amendments or comparable ethical standards. All of the information was anonymized, and the submission does not include images that might identify the enrolled patients in this study, according to published procedures.

### Immunohistochemistry for PD-L1, PD-1, Ki-67, and L1CAM

Formalin-fixed, paraffin-embedded (FFPE) samples from the 48 enrolled patients were retrospectively included in the study. The FFPE tissue slides from the 48 samples were immunostained by a two-step method. Anti-PD-L1 antibodies ((E1L3N) XP Rabbit mAb, 1:200; Cell Signaling Technology, Danvers, MA, USA), anti-PD-1 antibodies ((D4W2J) XP Rabbit mAb, 1:200; Cell Signaling Technology, Danvers, MA, USA), anti-L1CAM antibodies ((OTI10C12) XP Mouse mAb, 1:50; OriGene Technologies, Rockville, MD, USA), anti-Ki-67 antibodies ((MIB-1) XP Mouse mAb, 1:200; OriGene Wuxi Biotechnology Co, Wuxi, Jiangsu, CHN), and a DAB Detection Kit (Polymer) (PV-6000-D, ZSGB-BIO, Beijing, China) were used. Five fields were selected randomly in each case by ×400 magnification, and then the percentage of positive cells (PPC) and staining intensity (SI) were assessed using Image-pro Plus software, version 6.0 (Media Cybernetics, Inc., Rockville, MD, USA). The immunoreactive score (IRS) was determined based on the PPC and SI as follows: IRS = PPC × SI. The PPC scores were as follows: 0%, 0; 0–25%, 1; 25–50%, 2; 50–75%, 3; and 75–100%, 4. The SI scores were as follows: absent, 0; weak, 1; moderate, 2; and strong, 3. The expression levels of PD-L1, PD-1 and L1CAM were calculated as the mean IRSs of 5 fields, and Ki-67 was defined by the mean percentage of stained nuclei in 5 random fields.

### Statistical analysis

All analyses were conducted using IBM SPSS Statistics for Windows software (version 25.0). Univariate comparisons of PFS and OS were estimated using the Kaplan–Meier method, and significance testing (*α* = 0.05) was performed on the basis of the log-rank test. Multivariate Cox proportional hazards models were used to estimate the hazard ratios and 95% confidence intervals (CIs). Associations between the 3-year recurrence rate and PD-L1 expression were assessed using Pearson *χ*^2^ test, with *P* < 0.05 considered significant. Correlations of PD-L1, PD-1, L1CAM, and Ki-67 with WHO histologic grading were assessed by Spearman’s regression test, with *P* < 0.05 considered significant.

## Results

### Clinical features

Forty-eight patients were included in the study (23 male and 25 female). The ages of the patients in the study ranged from 1 to 61 years old (median, 16 years), and 21 patients (43.75%) were younger than 12 years of age. The duration of presenting symptoms ranged from 0.13 to 252 months, with an average of 13.50 months. According to the WHO classification system, 13 patients (27.08%) were grade II (ependymoma), and 35 (72.92%) were grade III (anaplastic ependymoma). GTR was achieved in 37 (77.08%) of the 48 patients, and STR was achieved in 11 (22.92%). Thirty patients (62.50%) underwent postoperative radiotherapy after the first operation, while 34 patients (70.83%) underwent radiotherapy during the course of the treatment. Twenty patients (41.67%) underwent postoperative chemotherapy, and 18 patients (37.50%) underwent concurrent radiotherapy and chemotherapy. The 3-, 5-, and 10-year PFS rates of patients without postoperative radiotherapy were 50.00% (3/6), 25.00% (1/4), and 0.00% (0/4), respectively, and that of patients with postoperative radiotherapy were 45.00% (9/20), 25.00% (4/16), and 8.33% (1/12), respectively, among patients with anaplastic ependymoma (WHO grade III).

Tumor recurrence occurred in 23 (47.92%) of the enrolled patients, including 18 patients with local tumor recurrence, 4 with distant metastasis, 1 with cerebrospinal dissemination, 3 with ependymoma (WHO grade II), and 20 with anaplastic ependymoma (WHO grade III). Among the 23 patients with recurrence, 15 patients received postoperative radiotherapy, and 8 patients received concurrent chemoradiotherapy. The 5- and 10-year recurrence rates were 75.00% (15/20) and 93.75% (15/16), respectively, in patients with anaplastic ependymoma and 50.00% (2/4) and 66.67% (2/3) in patients with ependymoma, respectively. The 5- and 10-year PFS rates of patients with anaplastic ependymoma (WHO grade III) were 25.00% (5/20) and 6.25% (1/16), respectively, and the 5- and 10-year OS rates were 45.00% (9/20) and 12.50% (2/16), respectively. The 5- and 10-year PFS rates of patients with ependymoma (WHO grade II) were 50.00% (2/4) and 33.33% (1/3), respectively, and the 5- and 10-year OS rates were 100.00% (4/4) and 100.00% (3/3), respectively. The 3-, 5- and 10-year PFS rates were 62.50% (15/24), 38.89% (7/18), and 15.38% (2/13), respectively, in patients with GTR. The 3-, 5-, and 10-year PFS rates were 75.00% (3/4), 50.00% (2/4), and 33.33% (1/3), respectively, in patients with ependymoma (WHO grade II) and GTR. The 3-, 5-, and 10-year PFS rates were 60.00% (12/20), 35.71% (5/14), and 10.00% (1/10), respectively, in patients with anaplastic ependymoma (WHO grade II) and GTR.

### Immunostaining of PD-L1, PD-1, Ki-67, and L1CAM

**Fig. 1:**
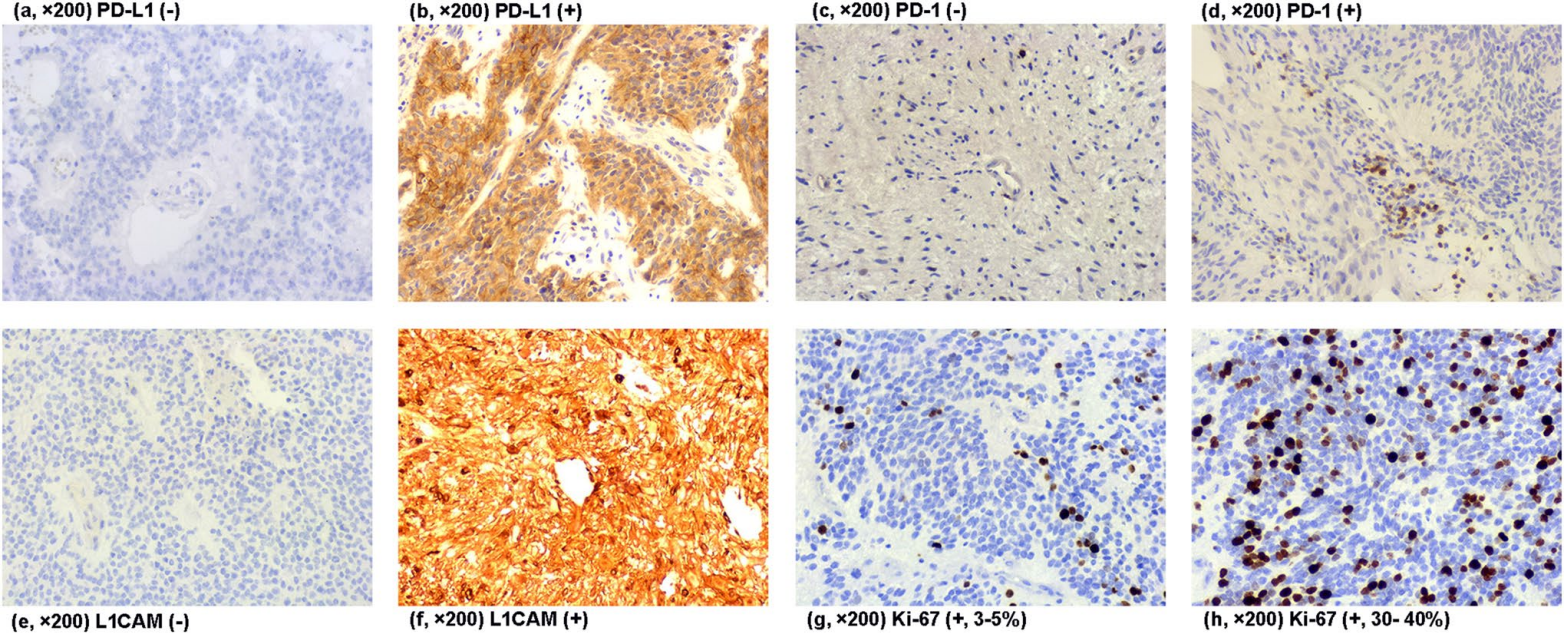
The expression of PD-L1, PD-1, L1CAM, and Ki-67 by immunohistochemistry. (**a**, × 200) Negative staining of PD-L1; (**b**, × 200) positive staining of PD-L1; (**c**, × 200) negative staining of PD-1; (**d**, × 200) positive staining of PD-1; (**e**, × 200) negative staining of L1CAM; (**f**, × 200) positive staining of L1CAM; (**g**, **h**, × 200) staining of Ki-67; **g** Ki-67-positive staining rate was 3–5%; **h** Ki-67-positive staining rate was 30–40%. *PD-L1* programmed death ligand-1, *PD-1* programmed cell death protein 1, *L1CAM* neural cell adhesion molecule L1

PD-L1 was positive in 21 (43.75%) tumor samples, while L1CAM was positive in 25 (52.08%) samples. PD-1 staining was positive in 18 (37.50%) tumor samples. The patterns of PD-L1, PD-1, L1CAM, and Ki-67 immunoreactivity are shown in Fig. 1.

### Prognostic factors

**Fig. 2:**
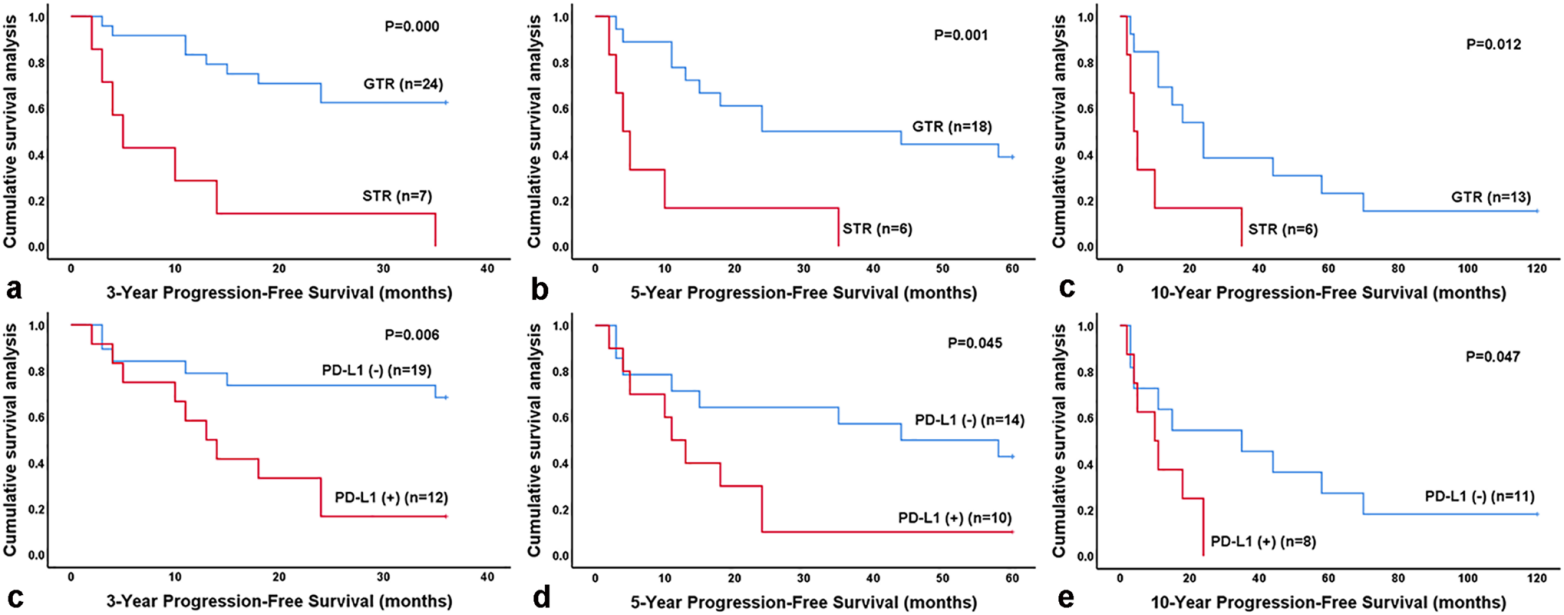
Kaplan–Meier analysis of progression-free survival for all patients. **a**–**c** Patients with GTR had significantly better progression-free survival than those with STR (*P* < 0.05); **d**–**f** patients with PD-L1 negativity had significantly better progression-free survival than those with PD-L1 positivity. GTR, gross total resection; STR, subtotal resection; PD-L1, programmed death ligand-1; PFS, progression-free survival

The results of the univariate analysis regarding clinical, therapeutic, and pathological prognostic factors are shown in Table 1, and the results of PD-L1, PD-1, Ki-67, and L1CAM analyses are shown in Table 2. PD-L1 expression and EOR reached significance in univariate analysis (*P* < 0.05; Tables 1 and 2) and were independent risk factors in multivariate Cox regression analysis (*P* < 0.05; Table 3). Patients with STR or PD-L1 positivity were associated with significantly worse PFS (*P* < 0.05; Fig. 2). No significant differences in survival by age, WHO histologic grade, radiotherapy, PD-1 and L1CAM were found, although trends toward improved survival were noted for ependymoma (WHO grade II) versus anaplastic ependymoma (WHO grade III) and low Ki-67 expression versus high expression.

**Table 1:** Patient characteristics and univariate survival analysis considering clinical, therapeutic and pathological prognostic factors (*n* = 48)

		3-year survival			5-year survival					10-year survival				
		*N*	PFS%	*P*	*N*	PFS %	*P*	OS%	*P*	*N*	PFS %	*P*	OS%	*P*
Age	< 12 years old	13	46.15	0.570	9	11.11	0.466	66.67	0.290	7	0.00	0.648	14.29	0.558
	≥ 12 years old	18	50.00		15	40.00		46.67		12	16.67		33.33	
Grade	II	5	60.00	0.391	4	50.00	0.154	100.00	0.079	3	33.33	0.145	100.00	0.019
	III	26	46.15		20	25.00		45.00		16	6.25		12.50	
Level of resection	GTR	24	62.50	0.000	18	38.89	0.001	61.11	0.128	13	15.38	0.012	38.46	0.146
	STR	7	0.00		6	0.00		33.33		6	0.00		0.00	
WHO grade II	GTR	4	75.00	0.046	4	50.00	NA	100.00	NA	3	33.33	NA	100.00	NA
	STR	1	0.00		0	NA		NA		0			NA	
WHO grade III	GTR	20	60.00	0.000	14	35.71	0.006	50.00	0.299	10	10.00	0.044	20.00	0.582
	STR	6	0.00		6	0.00		33.33		6	0.00		0.00	
Age < 12 years old	GTR	11	54.55	0.191	7	14.29	0.641	57.14	0.313	5	0.00	0.862	20.00	0.281
	STR	2	0.00		2	0.00		100.00		2	0.00		0.00	
Age ≥ 12 years old	GTR	13	69.23	0.000	11	54.55	0.000	63.64	0.004	8	25.00	0.001	50.00	0.026
	STR	5	0.00		4	0.00		0.00		4	0.00		0.00	
WHO grade III*	Without RT	6	50.00	0.894		*25.00* *(4)*	0.659	*0.00* *(3)*	0.104		*0.00* *(4)*	0.948	*0.00* *(3)*	0.301
	With RT	20	45.00			*25.00* *(16)*		*52.94* *(17)*			*8.33* *(12)*		*15.38* *(13)*	
WHO grade II and GTR	Without RT	3	66.67	0.564	3	66.67	0.515	100.00	NA	2	50.00	0.808	100.00	NA
	With RT	1	100.00		1	0.00		100.00		1	0.00		100.00	
WHO grade III and GTR	Without RT	5	60.00	0.779	3	66.67	0.741	33.33	0.430	3	0.00	0.974	33.33	0.770
	With RT	15	60.00		11	36.36		54.55		7	14.29		14.29	

*PFS* progression-free survival, *OS* overall survival, *GTR* gross total resection, *STR* subtotal resection, *RT* radiotherapy, *NA* not applicable, *N* the total number of patients with the corresponding follow-up time

*One patient with anaplastic ependymoma (WHO grade III) who did not receive radiotherapy after the first operation received radiotherapy after the second operation, resulting in a difference in the numbers of patients in the analyses of OS and PFS. Sixteen patients with RT were included in the 5-year PFS analysis, but 17 were included in the 5-year OS analysis. Twelve patients with RT were included in the 10-year PFS analysis, but 13 were included in the 10-year OS analysis. One more patient was counted by OS analysis than by PFS analysis in patients with RT. Four patients without RT were included in the 5-year PFS analysis, but 3 were included in the 5-year OS analysis. Four patients without RT were included in the 10-year PFS analysis, but 3 were included in the 10-year OS analysis. One more patient was counted by PFS analysis than by OS analysis in patients with RT

**Table 2:** Patient characteristics and univariate survival analysis considering PD-L1, PD-1, Ki-67 and L1CAM (*n* = 48)

		3-year survival			5-year survival					10-year survival				
		*N*	PFS %	*P*	*N*	PFS %	*P*	OS%	*P*	*N*	PFS %	*P*	OS%	*P*
PD-1	Negativity	19	57.89	0.368	16	37.50	0.309	62.50	0.357	12	16.67	0.764	33.33	0.551
	Positivity	12	33.33		8	12.50		37.50		7	0		14.29	
PD-L1	Negativity	19	68.42	0.006	14	42.86	0.045	64.29	0.245	11	18.18	0.047	36.36	0.191
	Positivity	12	16.67		10	10.00		40.00		8	0.00		12.50	
PD-L1 negative	GTR	16	81.25	0.001	11	54.55	0.013	72.73	0.259	8	25.00	0.058	50.00	0.248
	STR	3	0.00		3	0.00		33.33		3	0.00		0.00	
PD-L1 positive	GTR	8	25.00	0.024	7	14.29	0.012	42.86	0.185	5	0.00	0.046	20.00	0.268
	STR	4	0.00		3	0.00		33.33		3	0.00		0.00	
Age < 12 years old	PD-L1(−)	6	66.67	0.193	3	0.00	0.796	66.67	1.000	3	0.00	0.255	0.00	0.673
	PD-L1(+)	7	28.57		6	16.67		66.67		4	0.00		25.00	
Age ≥ 12 years old	PD-L1(−)	13	69.23	0.002	11	54.55	0.007	63.64	0.035	8	25.00	0.038	50.00	0.001
	PD-L1(+)	5	0.00		4	0.00		0.00		4	0.00		0.00	
GTR	PD-L1(−)	16	81.25	0.012	11	54.55	0.068	72.73	0.218	8	25.00	0.092	50.00	0.295
	PD-L1(+)	8	25.00		7	14.29		42.86		5	0.00		20.00	
PD-1(+) and PD-L1(+)	No	24	58.33	0.049	19	36.84	0.067	63.16	0.115	15	13.33	0.264	33.33	0.090
	Yes	7	14.29		5	0.00		20.00		4	0.00		0.00	
L1CAM	Negativity	19	52.63	0.685	13	30.77	0.727	46.15	0.299	11	18.18	0.776	36.36	0.742
	Positivity	12	41.67		11	27.27		63.64		8	0.00		12.50	
Ki-67 ≥ 20.5%	No	13	61.54	0.121	11	45.45	0.017	72.73	0.099	7	14.29	0.152	42.86	0.268
	Yes	18	38.89		13	16.67		38.46		12	8.33		16.67	
WHO grade II and GTR	PD-L1(−)	3	100.00	0.083	3	66.67	0.083	100.00	NA	2	50.00	0.157	100.00	NA
	PD-L1(+)	1	0.00		1	0.00		100.00		1	0.00		100.00	
WHO grade III and GTR	PD-L1(−)	13	76.92	0.056	8	50.00	0.243	62.50	0.393	6	16.67	0.294	33.33	0.228
	PD-L1(+)	7	28.57		6	16.67		33.33		4	0.00		0.00	

*PFS* progression-free survival, *OS* overall survival, *GTR* gross total resection, *STR* subtotal resection, *NA* not applicable, *N* the total number of patients with the corresponding follow-up time, (−) negative, (+) positive, *PD-L1* programmed death ligand-1, *PD-1* programmed cell death protein 1, *L1CAM* neural cell adhesion molecule L1

**Table 3:** Cox regression model for multivariate analysis (*n* = 48)

	3-year PFS			5-year PFS			10-year PFS		
	HR	95% CI	*P*	HR	95% CI	*P*	HR	95% CI	*P*
PD-L1 (positive vs. negative)	10.445	2.490–41.434	0.001	5.539	1.374–32.620	0.019	3.949	1.023–26.628	0.047
Level of resection (STR vs. GTR)	8.746	2.120–40.642	0.003	6.866	1.594–25.379	0.009	3.962	1.023–18.112	0.047
Grade (III vs. II)	1.338	0.412–31.297	0.247	0.026	0.077–8.786	0.871	0.021	0.118–11.930	0.886
L1CAM (positive vs. negative)	0.591	0.090–2.863	0.442	0.975	0.074–2.363	0.323	0.000	0.139–7.256	0.996
Ki-67 ≥ 20.5%	3.989	1.032–27.279	0.046	4.627	1.146–18.929	0.031	0.493	0.382–7.644	0.483
with RT vs. without RT	0.036	0.177–4.167	0.849	0.023	0.230–5.565	0.879	0.043	0.222–6.425	0.836
Age (< 12 years old vs. ≥ 12 years old)	2.971	0.829–18.609	0.085	0.452	0.309–11.056	0.501	0.898	0.430–11.267	0.343

*PFS* progression-free survival, *GTR* gross total resection, *STR* subtotal resection, *RT* radiotherapy, *PD-L1* programmed death ligand-1, *L1CAM* neural cell adhesion molecule L1, *HR* hazard ratio, *95% CI* 95% confidence interval

**Fig. 3:**
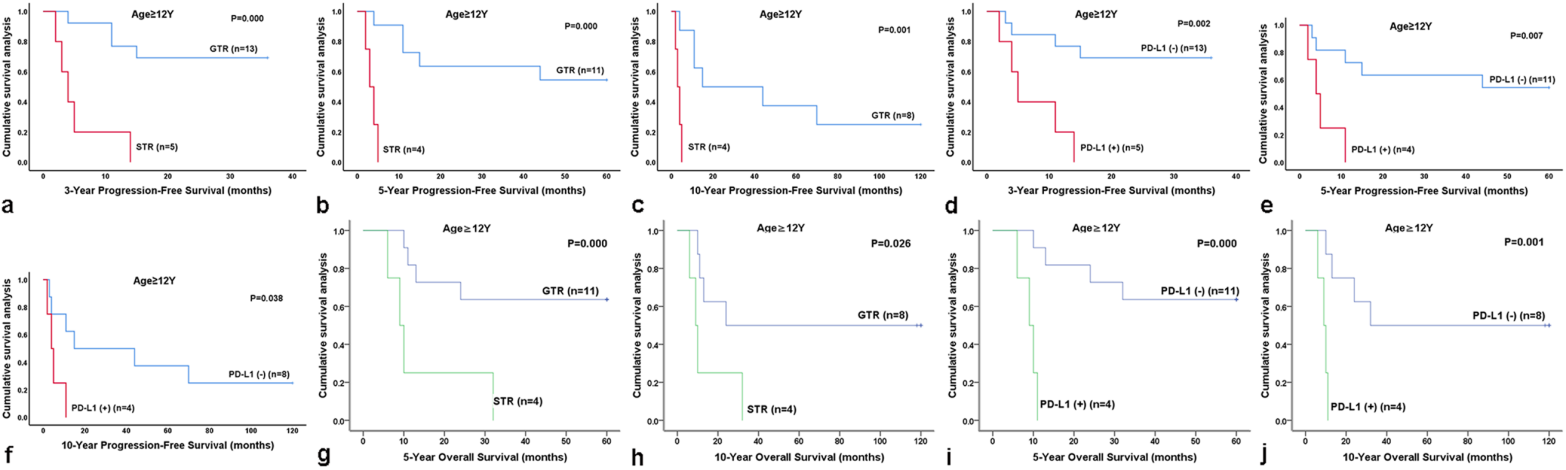
Kaplan–Meier analysis of survival for patients who were 12 years of age or older. **a**–**c** Patients with GTR had significantly better progression-free survival than those of patients with STR among patients 12 years of age or older (*P* < 0.05); **d**–**f** Patients with PD-L1 negativity had significantly better progression-free survival than those of patients with PD-L1 positivity among patients 12 years of age or older (*P* < 0.05); **g**, **h** Patients with GTR had significantly better overall survival than those of patients with STR among patients 12 years of age or older; **i**, **j** Patients with PD-L1 negativity had significantly better overall survival than those of patients with PD-L1 positivity among patients 12 years of age or older. *GTR* gross total resection, *STR* subtotal resection, *PD-L1* programmed death ligand-1, *PFS* progression-free survival, *OS* overall survival

**Fig. 4:**
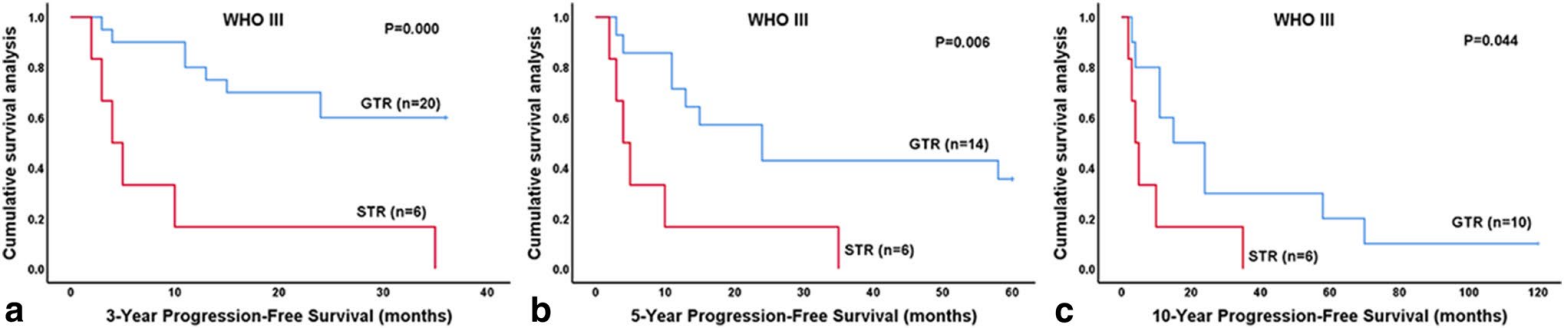
Kaplan–Meier analysis of progression-free survival for patients with anaplastic ependymoma (WHO grade III). **a**–**c** Patients with GTR had significantly better progression-free survival than those of patients with STR among patients with anaplastic ependymoma (WHO grade III) (*P* < 0.05). Because no patient with ependymoma (WHO grade II) and subtotal resection were followed up for more than 5 years, no survival curves of these patients are shown. *GTR* gross total resection, *STR* subtotal resection, *PFS* progression-free survival

Histologic grade and age are considered very important clinical prognostic factors; therefore, we conducted a progressive analysis of the effects of PD-L1 expression and EOR on the ependymoma (WHO grade II) and anaplastic ependymoma (WHO grade III) groups, as well as on the age groups ≥ 12 years and < 12 years. Notably, the PFS and OS times were longer in patients with GTR or PD-L1 negativity among patients 12 years of age or older (*P* < 0.05; Tables 1 and 2) (Fig. 3). Furthermore, STR was associated with worse PFS regardless of tumor grade (*P* < 0.05; Table 1) (Fig. 4).

Pearson *χ*^2^ test of prognostic factors suggested that only PD-L1 expression significantly correlated with the recurrence rate at 3 years (*κ* = 7.888, *P* = 0.005). The 3-year recurrence rate of patients with PD-L1 positivity was 83.3%, and the rate of patients with PD-L1 negativity was 31.6%, while Spearman’s correlation analysis of prognostic factors showed that only Ki-67 expression significantly correlated with WHO histologic grading (*r* = 0.637, *P* = 0.005).

## Discussion

The purpose of this study was to reveal the roles of clinical factors and immune checkpoint molecules in the prognosis of patients with SEE and to search for theoretical evidence for the treatment of these patients with immune checkpoint inhibitors.

Univariate Kaplan–Meier analysis of the effect of the EOR revealed that patients with GTR had longer 3-, 5- and 10-year PFS than those with STR, and the difference was significant (*P* < 0.05; Table 1). This finding suggests that total resection of the tumor can prolong the time of recurrence/progression and improve the PFS of these patients. The same conclusion was obtained when Cox multivariate regression analysis was performed and revealed that the recurrence rates of patients with STR at 3, 5, and 10 years were 8.746, 6.866 and 3.962 times those of patients with GTR, respectively (*P* < 0.005; Table 3). Kaplan–Meier analysis revealed that the PFS of the GTR subgroup was significantly better than that of the STR subgroup regardless of the histologic grade (*P* < 0.005; Table 1). This finding suggests that the EOR is an important independent predictive factor for tumor recurrence and progression in patients with SEE, and GTR should be performed regardless of the histologic grade to improve PFS and delay tumor recurrence. Our results also revealed that patients with GTR had significantly better PFS and OS than those of patients with STR among patients older than 12 years (*P* < 0.05; Table 1) (Fig. 3), while the difference among patients younger than 12 years was not significant (*P* > 0.05; Table 1). This outcome might have occurred because adult patients are better able to withstand the trauma caused by total surgical resection and thus enjoy the favorable outcomes associated with total resection. Therefore, our results suggest that GTR should be performed in patients 12 years of age or older to improve outcomes and delay tumor recurrence whenever feasible. Although 12 years of age may not be the exact age threshold, it is worth exploring whether the scope of surgical resection in pediatric patients can be appropriately reduced under the premise of total resection of the tumor as much as possible.

In recent years, Ki-67 has been the most studied prognostic immunohistological factor for patients with ependymoma (Figarella-Branger et al. 2007; Senetta et al. 2011; Milde et al. 2012; Chen et al. 2015; McLendon et al. 2015; Wostrack et al. 2018). Kuncova found that 18 of 67 factors reported in previous studies correlated with ependymoma progression, and only Ki-67 expression reached significance in predicting patients with ependymoma in a meta-analysis on the prognostic factors of ependymoma in 2007 (Kuncova et al. 2009). A higher expression level of Ki-67 corresponded to a worse outcome for these patients, but the cutoff value for a high expression level has varied greatly from study to study. In our study, Kaplan–Meier analysis was performed for Ki-67 expression according to the cutoff values mentioned in previous studies at 5.0%, 7.0%, 20.5%, and 25.0%. The results revealed that patients with Ki-67 < 20.5% had better PFS than those with Ki-67 ≥ 20.5%, although the difference was not significant (Table 2). This result was similar to the analysis results of WHO histologic grading; therefore, we conducted a correlation analysis between Ki-67 expression and histologic grading. The results showed that only the Ki-67 index significantly and positively correlated with WHO histologic grading when the correlations of the PD-L1, PD-1, L1CAM, Ki-67 indices with WHO histologic grading were analyzed by Spearman’s regression method (*r* = 0.637, *P* = 0.000). The Ki-67 protein is present during all active phases of the cell cycle (G1, S, G2, and mitosis) but is absent from resting cells (G0). Thus, Ki-67 is an excellent marker to determine the growth fraction of a given cell population. The higher the Ki-67 index, the more active the mitosis of ependymoma cells, the higher the cell proliferation, and the higher the WHO histologic grading, which is consistent with our results.

T-cell exhaustion induced by the PD-L1/PD-1 axis is one of the mechanisms by which tumors evade immune-mediated clearance (Pauken and Wherry 2015; Wherry and Kurachi 2015; Mirzaei et al. 2017). Previous studies have reported PD-L1 overexpression, increased CTL (CD8 + cytotoxic T lymphocyte) densities and T-cell exhaustion in RELA ependymoma and hypothesized that tumor evasion and immunosuppression occur due to T-cell exhaustion secondary to the interaction between PD-1 and PD-L1 (Hwang et al. 2018; Witt et al. 2018; Nambirajan et al. 2019). However, these studies did not verify the relationship between PD-L1 expression and the prognosis of ependymoma by survival analysis. Our study revealed that patients with PD-L1 negativity had significantly better PFS than those with PD-L1 positivity by Kaplan–Meier analysis (Table 2, *P* < 0.05) (Fig. 2). The Cox multivariate regression analysis yielded the same conclusion and revealed that the recurrence rates of patients with PD-L1 positivity at 3, 5, and 10 years were 10.445, 5.539, and 3.949 times those of patients with PD-L1 negativity, respectively (Table 3, *P* < 0.05). Pearson's Chi-square test was also performed and revealed that PD-L1 expression was significantly closely related to the 3-year recurrence rate (*κ* = 7.888 and *P* = 0.005). These results suggest that patients with PD-L1 positivity are more prone to relapse and have a shorter PFS time. Because age is believed to be a clinical prognostic marker in ependymoma, we studied PD-L1 expression in patients younger and older than 12 years old. Notably, in patients 12 years of age or older, the PFS and OS of patients with PD-L1 negativity were significantly better than those of patients with PD-L1 positivity (*P* < 0.05; Table 2) (Fig. 3), which indicates that patients 12 years of age or older with PD-L1 negativity can achieve better survival. In our study, the EOR and PD-L1 expression reached significance in the Kaplan–Meier univariate analysis and were independent of other factors in the Cox multivariate analysis (Tables 1, 2, 3), indicating that both factors could independently predict outcomes in patients with SEE. Moreover, the evaluation of PD-L1 expression was more readily obtained and assessed more accurately in predicting the outcomes of these patients.

The 3-, 5-, and 10-year PFS rates were 62.50% (15/24), 38.89% (7/18), and 15.38% (2/13), respectively, in patients with GTR. This result revealed that total resection of the tumor during surgery could not completely prevent tumor recurrence; therefore, our study analyzed the PFS times of patients with GTR. The result revealed that patients with PD-L1 negativity had longer 3-year PFS than patients with PD-L1 positivity, with a difference that was significant (*P* = 0.012; Table 2) by Kaplan–Meier analysis. The patients with PD-L1 negativity had longer 5- and 10-year PFS than patients with PD-L1 positivity in the survival curve, although the difference was not significant (*P* = 0.068, *P* = 0.092; Table 2). Furthermore, the estimated mean PFS time and rates of patients with PD-L1 negativity were better than those of patients with PD-L1 positivity. The same trend was found in the PFS and OS analysis of patients with anaplastic ependymoma (WHO grade III) and the PFS analysis of patients with ependymoma (WHO grade II) (Table 2). PD-L1 inhibitors can enhance the body’s anti-tumor immunity by inhibiting T-cell exhaustion caused by the PD-L1/PD-1 axis or other mechanisms, and they have shown to be an effective method for the treatment of a series of tumors, including various intracranial tumors. Our results suggested that PD-L1 inhibitors might be an option for postoperative adjuvant therapy in these patients and might be useful for survival improvement in patients with SEE after the tumor has been completely removed. PD-L1 inhibitors could be a more effective alternative to conventional radiotherapy and chemotherapy for patients with SEE.

Histopathological diagnosis, according to the WHO classification system, is an important basis for clinical work (Ellison et al. 2011; Gajjar et al. 2015; Pajtler et al. 2017) but remains controversial in predicting the outcomes of patients with ependymomas (Guyotat et al. 2009; Kilday et al. 2009; Vera-Bolanos et al. 2015; Byun et al. 2018; Snider et al. 2018). This study revealed the 10-year OS of patients with ependymoma (WHO grade II) was significantly longer than those with anaplastic ependymoma (WHO grade III) (*P* < 0.05; Table 1), although the differences in PFS and 5-year OS were not significant. These results indicate that the effect of WHO histologic grading alone on the prognosis of patients with SEE remains limited, and further study is needed. However, the WHO classification for CNS tumors is still a very reasonable diagnostic basis for such patients, and the continuous improvement of its biological characteristics can lead to a more accurate determination of the grading of such patients, especially when distinguishing between ependymoma (WHO grade II) and anaplastic ependymoma (WHO grade III).

The role of chemotherapy for the treatment of patients with ependymoma remains unclear and is considered only when local treatment options (surgery and radiotherapy) have been exhausted. Although the efficacy of radiotherapy is controversial, it remains the primary adjuvant therapy, especially for patients with anaplastic ependymoma (Vera-Bolanos et al. 2015; Rudà et al. 2018). In this study, the 3-, 5-, and 10-year PFS rates of patients with anaplastic ependymoma (WHO III) were analyzed, with no difference in PFS time between patients receiving postoperative radiotherapy and those receiving no postoperative radiotherapy (*P* > 0.05; Table 1). The results showed that postoperative radiotherapy did not significantly improve PFS in patients with anaplastic ependymoma in the univariate analysis. Although the difference in OS was not significant between patients receiving radiotherapy and those receiving no radiotherapy, the OS rate, and time of former patients receiving radiotherapy were better than those of patients who did not (Table 1). In conclusion, although radiotherapy cannot improve the PFS times of patients with anaplastic ependymoma (WHO III), it can extend the total survival time of these patients.

The 3-, 5-, and 10-year PFS rates were 75.00% (3/4), 50.00% (2/4), and 33.33% (2/3), respectively, in patients with ependymoma (WHO grade II) and GTR, indicating that total resection could not completely prevent tumor recurrence in patients with ependymoma (WHO grade II); thus, our study analyzed PFS among patients with ependymoma and GTR with or without postoperative radiotherapy. The 3-, 5-, and 10-year PFS rates were 66.77% (2/3), 66.77% (2/3), and 50.00% (1/2), respectively, in patients without postoperative radiotherapy, and the 3-, 5-, and 10-year PFS rates were 100.00% (1/1), 0.00% (0/1), and 0.00% (0/1), respectively, in patients with postoperative radiotherapy. The results revealed that postoperative radiotherapy did not have prognostic significance in patients with ependymoma (WHO grade II) and GTR (*P* > 0.05; Table 1). One patient without postoperative radiotherapy relapsed 18 months after surgery, and one patient with postoperative radiotherapy relapsed 44 months after surgery. Both patients received radiotherapy after the second operation, and no recurrence occurred after 10 years of follow-up. This treatment process is consistent with the treatment principles of intracranial ependymoma in the guidelines of the NCCN (National Comprehensive Cancer Network) and the EANO (European Association for Neuro-Oncology) (Rudà et al. 2018). Further studies are needed to determine whether radiotherapy should be performed after total resection in patients with ependymoma (WHO grade II). Our results also revealed that postoperative radiotherapy did not have prognostic significance in patients with anaplastic ependymoma (WHO grade III) after the tumor was completely removed (*P* > 0.05; Table 1). However, patients with recurrent ependymomas should receive secondary total surgical resection and radiotherapy (Byun et al. 2018; Rudà et al. 2018).

In our study, PD-1 expression did not have prognostic significance in patients with SEE; however, patients with PD-1 negativity had better PFS and OS rates than those with PD-1 positivity (Table 2, *P* > 0.05). The same trend was found among patients with and without both positive PD-L1 and PD-1 (Table 2, *P* > 0.05). These results are consistent with the idea of T-cell exhaustion caused by the PD-L1/PD-1 axis. However, there are multiple factors and mechanisms that cause lymphocyte exhaustion, and PD-L1 might cause lymphocyte exhaustion by combining with various ligands. Therefore, further studies of lymphocyte exhaustion and the role of PD-1 in SEE are needed.

Several studies have shown that the outcomes of patients with C11orf95-RELA fusion-positive ependymoma are worse than those of patients with the other two subtypes (Pajtler et al. 2015; Malgulwar et al. 2018; Wang et al. 2019). Malgulwar et al. (2018) and Wang et al. (2019) found that L1CAM was consistent with the RELA fusion gene by more than 80%. In our study, amazingly, the PFS and OS of all patients were analyzed by Kaplan–Meier and Cox analyses, which revealed no difference between patients with L1CAM positivity and patients with L1CAM negativity (*P* > 0.05; Tables 2 and 3). Our results also showed no correlation between L1CAM expression and WHO histologic grading by the Spearman regression method (*r* = 0.053, *P* = 0.721). The reason for this inconsistent result might be the poor consistency of the application of L1CAM alone in the detection of the C11orf95-RELA fusion gene when we analyzed the data from our study and the recent literature. Several studies have suggested that markers such as P65 could also be included in tests to assess the presence of the C11orf95-RELA fusion gene (Gessi et al. 2019; Wang et al. 2019). In addition, although the presence of the RELA fusion gene is associated with poor outcomes, the difference might not be significant. Furthermore, although EPN variation at different anatomical sites has histopathological similarities, its molecular biology is heterogeneous. Further research is needed regarding how to detect the presence of the C11orf95-RELA fusion gene more accurately and to determine the role of L1CAM in predicting the outcomes of patients with SEE, especially at present, when genetic testing is limited in clinical work.

The role of age in predicting the outcomes of patients with intracranial ependymoma is controversial (Kilday et al. 2009; Sun et al. 2018). In our study, age did not have prognostic significance in patients with SEE (Tables 1 and 3). Much of the efficacy of age found in other studies could be contributed to the tumor location and the EOR. Most ependymomas in children occur in the posterior fossa and are mostly removed subtotally, while most ependymomas in adults occur in the supratentorial location and are easily completely removed. Subsequently, the patients were divided into a group of children younger than 12 years of age and an adult group of those 12 years of age or older for statistical analysis. In patients 12 years old or older, PD-L1 and EOR had prognostic significance (*P* < 0.05; Table 2), while PD-1, L1CAM, Ki-67, and WHO histologic grading had no prognostic significance. In addition, the expression of PD-L1, PD-1, L1CAM, and Ki-67, WHO histologic grading and EOR had no prognostic significance in patients younger than 12 years of age.

This study has the classic limitations of a retrospective analyses, which include the short follow-up time of some cases. In this study, the enrolled patients were grouped according to the follow-up time in the corresponding survival analysis to guarantee that the conclusions were correct and reliable. Furthermore, the reliability of the results and the stability of the statistical model were verified by different statistical methods. However, it is still necessary to enlarge the sample size and extend the follow-up time for further analysis.

The different prognostic perspectives regarding WHO histological grade might be due to poor interobserver reproducibility and/or high histological heterogeneity. Therefore, another obstacle was determining histological grade according to the WHO criteria. In this study, one enrolled patient was diagnosed with meningioma at the first operation but anaplastic ependymoma at the second operation after recurrence. The first operative specimen was re-examined and diagnosed as anaplastic ependymoma, which revealed the aforementioned problem. In our study, all of the enrolled patients were rescreened by the same senior neuropathologist to confirm their diagnosis and histological grade.

## Conclusions

We believe that PD-L1 immunostaining is a reproducible and reliable method with strong ability to predict the outcomes of patients with SEE. Moreover, PD-L1 expression was more readily obtained and assessed more accurately in predicting the outcomes of these patients. Additionally, PD-L1 inhibitors could be an option for the treatment of patients with SEE and an even more effective alternative to radiotherapy for patients with anaplastic ependymoma after the tumor has been completely removed. A more complete characterization of the SEEs coupled with their immunogenicity would potentially allow for more targeted immune therapies against subsets of these tumors. Particularly for children under 3 years who cannot receive radiotherapy, PD-L1-related immune checkpoint inhibitors may be able to delay tumor recurrence to buy time for radiotherapy and further improve the outcomes of these patients.

## Data Availability

The datasets generated during and/or analyzed during the current study are available from the corresponding author on reasonable request.

## Code availability

All the statistical analyses and survival curve were performed using SPSS 25.0 software (IBM Corporation, Chicago, IL, USA).

## References

[CR1] Byun J, Kim JH, Kim YH, Cho YH, Hong SH, Kim CJ (2018) Supratentorial extraventricular ependymoma: retrospective analysis of 15 patients at a single institution. World Neurosurg 118:e1–e9. 10.1016/j.wneu.2018.05.192 29879510 10.1016/j.wneu.2018.05.192

[CR2] Chen L, Zou X, Wang Y, Mao Y, Zhou L (2013) Central nervous system tumors: a single center pathology review of 34,140 cases over 60 years. BMC Clin Pathol 13:14. 10.1186/1472-6890-13-14 23639346 10.1186/1472-6890-13-14 PMC3655926

[CR3] Chen X, Li C, Che X, Chen H, Liu Z (2015) Spinal myxopapillary ependymomas: a retrospective clinical and immunohistochemical study. Acta Neurochir 158:101–107. 10.1007/s00701-015-2637-8 26577638 10.1007/s00701-015-2637-8

[CR4] Ellison DW, Kocak M, Figarella-Branger D, Felice G, Catherine G, Pietsch T, Frappaz D, Massimino M, Grill J, Boyett JM, Grundy RG (2011) Histopathological grading of pediatric ependymoma: reproducibility and clinical relevance in European trial cohorts. J Negat Results Biomed 10:7. 10.1186/1477-5751-10-7 21627842 10.1186/1477-5751-10-7 PMC3117833

[CR5] Figarella-Branger D, Metellus P, Barrié M et al (2007) Épendymomes intracrâniens de l’adulte. Diagnostic histologique et facteurs histopronostiques. Neurochirurgie 53:76–84. 10.1016/j.neuchi.2006.11.007 17445840 10.1016/j.neuchi.2006.11.007

[CR6] Gajjar A, Bowers DC, Karajannis MA, Leary S, Witt H, Gottardo NG (2015) Pediatric brain tumors: innovative genomic information is transforming the diagnostic and clinical landscape. J Clin Oncol 33:2986–2998. 10.1200/JCO.2014.59.9217 26304884 10.1200/JCO.2014.59.9217 PMC4567701

[CR7] Gessi M, Giagnacovo M, Modena P et al (2019) Role of immunohistochemistry in the identification of supratentorial C11ORF95-RELA fused ependymoma in routine neuropathology. Am J Surg Pathol 43:56–63. 10.1097/pas.0000000000000979 29266023 10.1097/PAS.0000000000000979

[CR8] Guyotat J, Metellus P, Giorgi R, Barrie M, Jouvet A, Fevre-Montange M, Chinot O, Durand A, Figarella-Branger D (2009) Infratentorial ependymomas: prognostic factors and outcome analysis in a multi-center retrospective series of 106 adult patients. Acta Neurochir 151:947–960. 10.1007/s00701-009-0417-z 19499166 10.1007/s00701-009-0417-z

[CR9] Hwang K, Koh EJ, Choi EJ et al (2018) PD-1/PD-L1 and immune-related gene expression pattern in pediatric malignant brain tumors: clinical correlation with survival data in Korean population. J Neuro-Oncol 139:281–291. 10.1007/s11060-018-2886-5 10.1007/s11060-018-2886-5 29730815

[CR10] Jung TY, Jung S, Kook H, Baek HJ (2018) Treatment decisions of world health organization grade II and III ependymomas in molecular era. J Korean Neurosurg Soc 61:312–318. 10.3340/jkns.2018.0003 29742878 10.3340/jkns.2018.0003 PMC5957310

[CR11] Kilday JP, Rahman R, Dyer S, Ridley L, Lowe J, Coyle B, Grundy R (2009) Pediatric ependymoma: biological perspectives. Mol Cancer Res 7:765–786. 10.1158/1541-7786.mcr-08-0584 19531565 10.1158/1541-7786.MCR-08-0584

[CR12] Kuncova K, Janda A, Kasal P, Zamecnik J (2009) Immunohistochemical prognostic markers in intracranial ependymomas: systematic review and meta-analysis. Pathol Oncol Res 15:605–614. 10.1007/s12253-009-9160-2 19301151 10.1007/s12253-009-9160-2

[CR13] Leng X, Tan X, Zhang C, Lin H, Qiu S (2016) Magnetic resonance imaging findings of extraventricular anaplastic ependymoma: a report of 11 cases. Oncol Lett 12:2048–2054. 10.3892/ol.2016.4825 27602137 10.3892/ol.2016.4825 PMC4998574

[CR14] Lin FY, Chintagumpala M (2015) Advances in management of pediatric ependymomas. Curr Oncol Rep 17:47. 10.1007/s11912-015-0470-0 26369328 10.1007/s11912-015-0470-0

[CR15] Malgulwar PB, Nambirajan A, Pathak P, Faruq M, Rajeshwari M, Singh M, Suri V, Sarkar C, Sharma MC (2018) C11orf95-RELA fusions and upregulated NF-KB signalling characterise a subset of aggressive supratentorial ependymomas that express L1CAM and nestin. J Neuro-Oncol 138:29–39. 10.1007/s11060-018-2767-y 10.1007/s11060-018-2767-y 29354850

[CR16] McLendon RE, Lipp E, Satterfield D et al (2015) Prognostic marker analysis in pediatric intracranial ependymomas. J Neuro-Oncol 122:255–261. 10.1007/s11060-014-1711-z 10.1007/s11060-014-1711-z 25563815

[CR17] Merchant TE, Li C, Xiong X, Kun LE, Boop FA, Sanford RA (2009) Conformal radiotherapy after surgery for paediatric ependymoma: a prospective study. Lancet Oncol 10:258–266. 10.1016/s1470-2045(08)70342-5 19274783 10.1016/S1470-2045(08)70342-5 PMC3615425

[CR18] Metellus P, Barrie M, Figarella-Branger D, Chinot O, Giorgi R, Gouvernet J, Jouvet A, Guyotat J (2007) Multicentric French study on adult intracranial ependymomas: prognostic factors analysis and therapeutic considerations from a cohort of 152 patients. Brain 130:1338–1349. 10.1093/brain/awm046 17449478 10.1093/brain/awm046

[CR19] Milde T, Hielscher T, Witt H et al (2012) Nestin expression identifies ependymoma patients with poor outcome. Brain Pathol 22:848–860. 10.1111/j.1750-3639.2012.00600.x 22568867 10.1111/j.1750-3639.2012.00600.x PMC8057643

[CR20] Mirzaei R, Sarkar S, Yong VW (2017) T cell exhaustion in glioblastoma: intricacies of immune checkpoints. Trends Immunol 38:104–115. 10.1016/j.it.2016.11.005 27964820 10.1016/j.it.2016.11.005

[CR21] Nambirajan A, Malgulwar PB, Sharma A, Boorgula MT, Doddamani R, Singh M, Suri V, Sarkar C, Sharma MC (2019) Clinicopathological evaluation of PD-L1 expression and cytotoxic T-lymphocyte infiltrates across intracranial molecular subgroups of ependymomas: are these tumors potential candidates for immune check-point blockade? Brain Tumor Pathol 36:152–161. 10.1007/s10014-019-00350-1 31388782 10.1007/s10014-019-00350-1

[CR22] Pajtler KW, Witt H, Sill M et al (2015) Molecular classification of ependymal tumors across all CNS compartments, histopathological grades, and age groups. Cancer Cell 27:728–743. 10.1016/j.ccell.2015.04.002 25965575 10.1016/j.ccell.2015.04.002 PMC4712639

[CR23] Pajtler KW, Mack SC, Ramaswamy V et al (2017) The current consensus on the clinical management of intracranial ependymoma and its distinct molecular variants. Acta Neuropathol 133:5–12. 10.1007/s00401-016-1643-0 27858204 10.1007/s00401-016-1643-0 PMC5209402

[CR24] Pauken KE, Wherry EJ (2015) Overcoming T cell exhaustion in infection and cancer. Trends Immunol 36:265–276. 10.1016/j.it.2015.02.008 25797516 10.1016/j.it.2015.02.008 PMC4393798

[CR25] Pejavar S, Polley MY, Rosenberg-Wohl S, Chennupati S, Prados MD, Berger MS, Banerjee A, Gupta N, Haas-Kogan D (2011) Pediatric intracranial ependymoma: the roles of surgery, radiation and chemotherapy. J Neuro-Oncol 106:367–375. 10.1007/s11060-011-0671-9 10.1007/s11060-011-0671-9 21826561

[CR26] Pérez-Ramírez M, García-Méndez A, Siordia-Reyes AG, Chavarría A, Gómez C, García-Hernández N (2019) Pediatric ependymoma: GNAO1, ASAH1, IMMT and IPO7 protein expression and 5-year prognosis correlation. Clin Neurol Neurosurg 186:105488. 10.1016/j.clineuro.2019.105488 31505435 10.1016/j.clineuro.2019.105488

[CR27] Ramaswamy V, Taylor MD (2016) Treatment implications of posterior fossa ependymoma subgroups. Chin J Cancer 35:93. 10.1186/s40880-016-0155-6 27846874 10.1186/s40880-016-0155-6 PMC5111181

[CR28] Reni M, Mazza E, Zanon S, Gatta G, Vecht CJ (2017) Central nervous system gliomas. Crit Rev Oncol/Hematol 113:213–234. 10.1016/j.critrevonc.2017.03.021 28427510 10.1016/j.critrevonc.2017.03.021

[CR29] Rudà R, Reifenberger G, Frappaz D, Pfister SM, Laprie A, Santarius T, Roth P, Tonn JC, Soffietti R, Weller M, Moyal ECJ (2018) EANO guidelines for the diagnosis and treatment of ependymal tumors. Neuro-oncology 20:445–456. 10.1093/neuonc/nox166 29194500 10.1093/neuonc/nox166 PMC5909649

[CR30] Sato M, Gunther JR, Mahajan A et al (2017) Progression-free survival of children with localized ependymoma treated with intensity-modulated radiation therapy or proton-beam radiation therapy. Cancer 123:2570–2578. 10.1002/cncr.30623 28267208 10.1002/cncr.30623

[CR31] Sayegh ET, Aranda D, Kim JM, Oh T, Parsa AT, Oh MC (2014) Prognosis by tumor location in adults with intracranial ependymomas. J Clin Neurosci 21:2096–2101. 10.1016/j.jocn.2014.05.011 25037313 10.1016/j.jocn.2014.05.011 PMC4474735

[CR32] Senetta R, Miracco C, Lanzafame S, Chiusa L, Caltabiano R, Galia A, Stella G, Cassoni P (2011) Epidermal growth factor receptor and caveolin-1 coexpression identifies adult supratentorial ependymomas with rapid unfavorable outcomes. Neuro-oncology 13:176–183. 10.1093/neuonc/noq160 21059755 10.1093/neuonc/noq160 PMC3064620

[CR33] Snider CA, Yang K, Mack SC, Suh JH, Chao ST, Merchant TE, Murphy ES (2018) Impact of radiation therapy and extent of resection for ependymoma in young children: a population-based study. Pediatr Blood Cancer 65:e26880. 10.1002/pbc.26880 10.1002/pbc.26880 29115718

[CR34] Sun S, Wang J, Zhu M, Beejadhursing R, Gao P, Zhang X, Jiao L, Jiang W, Ke C, Shu K (2018) Clinical, radiological, and histological features and treatment outcomes of supratentorial extraventricular ependymoma: 14 cases from a single center. J Neurosurg 128:1396–1402. 10.3171/2017.1.jns161422 28686116 10.3171/2017.1.JNS161422

[CR35] Svoboda N, Bradac O, de Lacy P, Benes V (2018) Intramedullary ependymoma: long-term outcome after surgery. Acta Neurochir 160:439–447. 10.1007/s00701-017-3430-7 29280009 10.1007/s00701-017-3430-7

[CR36] Tarapore PE, Modera P, Naujokas A, Oh MC, Amin B, Tihan T, Parsa AT, Ames CP, Chou D, Mummaneni PV, Weinstein PR (2013) Pathology of spinal ependymomas. Neurosurgery 73:247–255. 10.1227/01.neu.0000430764.02973.78 23670032 10.1227/01.neu.0000430764.02973.78

[CR37] Venkatramani R, Ji L, Lasky J et al (2013) Outcome of infants and young children with newly diagnosed ependymoma treated on the “Head Start” III prospective clinical trial. J Neuro-Oncol 113:285–291. 10.1007/s11060-013-1111-9 10.1007/s11060-013-1111-9 PMC4119804 23508296

[CR38] Vera-Bolanos E, Aldape K, Yuan Y et al (2015) Clinical course and progression-free survival of adult intracranial and spinal ependymoma patients. Neuro-oncology 17:440–447. 10.1093/neuonc/nou162 25121770 10.1093/neuonc/nou162 PMC4483095

[CR39] Wang M, Zhang R, Liu X, Li D, Qiu C, Zhao P, Zuo Y, Zhang P, Wang J, Sun H (2018) Supratentorial extraventricular ependymomas: a retrospective study focused on long-term outcomes and prognostic factors. Clin Neurol Neurosurg 165:1–6. 10.1016/j.clineuro.2017.12.013 29253745 10.1016/j.clineuro.2017.12.013

[CR40] Wang L, Liu L, Li H et al (2019) RELA fusion in supratentorial extraventricular ependymomas: a morphologic, immunohistochemical, and molecular study of 43 cases. Am J Surg Pathol 43:1674–1681. 10.1097/pas.0000000000001342 31393268 10.1097/PAS.0000000000001342

[CR41] Wherry EJ, Kurachi M (2015) Molecular and cellular insights into T cell exhaustion. Nat Rev Immunol 15:486–499. 10.1038/nri3862 26205583 10.1038/nri3862 PMC4889009

[CR42] Witt DA, Donson AM, Amani V, Moreira DC, Sanford B, Hoffman LM, Handler MH, Levy JMM, Jones KL, Nellan A, Foreman NK, Griesinger AM (2018) Specific expression of PD-L1 in RELA-fusion supratentorial ependymoma: implications for PD-1-targeted therapy. Pediatr Blood Cancer 65:e26960. 10.1002/pbc.26960 29350470 10.1002/pbc.26960 PMC5867234

[CR43] Wostrack M, Ringel F, Eicker SO et al (2018) Spinal ependymoma in adults: a multicenter investigation of surgical outcome and progression-free survival. J Neurosurg Spine 28:654–662. 10.3171/2017.9.spine17494 29521579 10.3171/2017.9.SPINE17494

[CR44] Ye J, Zhu J, Yan J, Chen P, Wan Z, Chen F, Zhang L, Qian J, Luo C (2015) Analysis on therapeutic outcomes and prognostic factors of intracranial ependymoma: a report of 49 clinical cases in a single center. Neurol Sci 36:2253–2261. 10.1007/s10072-015-2347-2 26216494 10.1007/s10072-015-2347-2

